# Mitral Valve Regurgitation: A Severe Complication following Left Ventricular Biopsy 15 Years after Heart Transplantation

**DOI:** 10.1155/2013/407875

**Published:** 2013-03-11

**Authors:** Marcel Vollroth, Joerg Seeburger, Philipp Kiefer, Jens Garbade, Friedrich W. Mohr, Markus J. Barten

**Affiliations:** Department of Cardiac Surgery, Heartcenter Leipzig University, Struempellstraße 39, 04289 Leipzig, Germany

## Abstract

A 71-year-old male patient underwent orthotopic heart transplantation in 1995. Due to left heart catheterization 15 years later, biopsy from the left ventricular apex was performed for rejection screening. Two days later, echocardiography revealed severe mitral valve regurgitation requiring mitral valve replacement. This is a very rare case showing that left heart biopsy may lead to severe hemodynamic complications with the need for surgical intervention.

## 1. Introduction

In patients after orthotopic heart transplantation, endomyocardial biopsy (EMB) represents a well-established technique for followup and diagnosis of histopathologic alterations before allograft dysfunction [1, 2]. Percutaneous transvenous EMB from the right ventricle remains the gold standard. However, it is an invasive procedure with potential morbidity of ventricular perforation, pneumothorax, and tricuspid valve injury [3]. In combination with coronary catheterization for detection of allograft vasculopathy, left heart biopsy appears as a reasonable procedure. Hereby, we describe a case of severe mitral valve regurgitation following transarterial left heart biopsy 15 years after orthotopic heart transplantation.

## 2. Case Presentation

A 71-year-old man underwent orthotopic heart transplantation in 1995 because of severe ischemic cardiomyopathy. The postoperative course was uneventful, and he was discharged to rehabilitation 18 days later. During the following 14 years, myocardial biopsy was performed from the right ventricle without any complications. None of them showed a significant rejection. In 2010, the patient became symptomatic with dyspnoea and arrhythmias during physical activity. A left heart catheterization was performed to detect any coronary alterations. We found only mild transplant vasculopathy without significant stenosis, a good ejection fraction, and patent valves. During this procedure, we took four biopsy samples from the left ventricular apex to detect significant rejection. Subsequently, the patient was transferred to transplant ward in a stable condition. Microscopic examination of the biopsy samples showed no signs of rejection.

**Figure 1:**
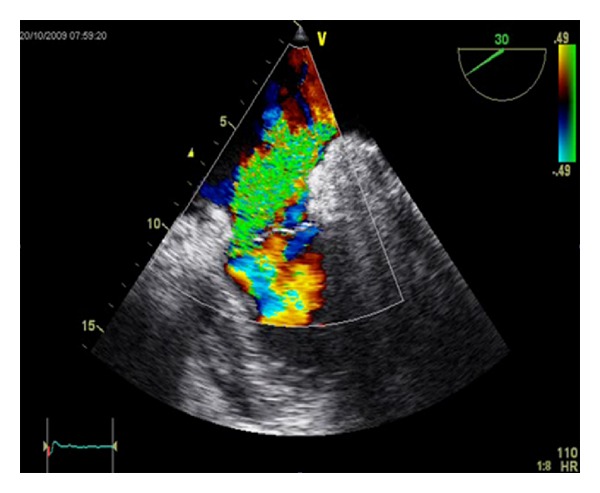
Echocardiogram shows severe mitral valve regurgitation.

Two days after the intervention, the patient presented severe dyspnoea according to NYHA Class IV and bilateral pleural effusion. A transthoracic echocardiogram detected an enlarged left atrium and severe mitral valve regurgitation with posterior leaflet prolapse (Figure 1). 

Our heart team decided for mitral valve operation one day later. Via full sternotomy and in mild hypothermia during cardiopulmonary bypass, the patient achieved mitral valve replacement by a 31 mm St. Jude biological valve. Intraoperative findings showed multiple clefts in the posterior leaflet and severely damaged cords. 

The postoperative course was uneventful, and the patient was discharged on the 14th postoperative day. Two years after the operation, the patient is in a very good condition.

## 3. Discussion

With this case, we report a fatal complication after left heart endomyocardial biopsy. The right ventricular access is widely accepted and has a low incidence of complications. The mortality ranges between 0% and 0.4% [3]. In combination with left heart catheterization, left heart biopsy appears reasonable. However, the hemodynamic consequence of mitral valve injury due to the bioptome leads to severe cardio pulmonary symptoms with the need for mitral valve repair or replacement. 

A valve replacement in a transplanted heart is a very rare procedure and should be avoided by all means [4].

## 4. Conclusion

We think that left heart biopsy is very unsafe and compromises patients' life if mitral valve injury happens. EMB from the right ventricle represents the approach of choice for cardiac rejection screening. This procedure is very safe with very low complication rates.
